# Climate change reduces nectar secretion in two common Mediterranean plants

**DOI:** 10.1093/aobpla/plv111

**Published:** 2015-09-15

**Authors:** Krista Takkis, Thomas Tscheulin, Panagiotis Tsalkatis, Theodora Petanidou

**Affiliations:** Laboratory of Biogeography and Ecology, Department of Geography, University of the Aegean, University Hill, GR-81100 Mytilene, Greece

**Keywords:** *Ballota acetabulosa*, elevated temperatures, global change, nectar production, nectar sugar content, phrygana, plant–pollinator interactions, *Teucrium divaricatum*

## Abstract

Global warming can lead to considerable impacts on natural plant communities, potentially inducing changes in plant physiology and the quantity and quality of floral rewards, especially nectar. Changes in nectar production can in turn strongly affect plant–pollinator interaction networks—pollinators may potentially benefit under moderate warming conditions, but suffer as resources reduce in availability as elevated temperatures become more extreme. Here, we studied the effect of elevated temperatures on nectar secretion of two Mediterranean Lamiaceae species—*Ballota acetabulosa* and *Teucrium divaricatum*. We measured nectar production (*viz*. volume per flower, sugar concentration per flower and sugar content per flower and per plant), number of open and empty flowers per plant, as well as biomass per flower under a range of temperatures selected *ad hoc* in a fully controlled climate chamber and under natural conditions outdoors. The average temperature in the climate chamber was increased every 3 days in 3 °C increments from 17.5 to 38.5 °C. Both study species showed a unimodal response of nectar production (volume per flower, sugar content per flower and per plant) to temperature. Optimal temperature for sugar content per flower was 25–26 °C for *B. acetabulosa* and 29–33 °C for *T. divaricatum*. According to our results, moderate climate warming predicted for the next few decades could benefit nectar secretion in *T. divaricatum* as long as the plants are not water stressed, but have a moderate negative effect on *B. acetabulosa*. Nevertheless, strong warming as predicted by climate change models for the end of the 21st century is expected to reduce nectar secretion in both species and can thus significantly reduce available resources for both wild bees and honeybees in Mediterranean systems.

## Abstract (precis)

The negative effect of climate change on plants and plant–pollinator interactions is a matter of concern worldwide. The Mediterranean region is considered particularly susceptible to climate warming and the communities of this region will need to face considerable climatic changes over the 21^st^ century. We studied the effect of temperature on nectar secretion of two Mediterranean species (*Ballota acetabulosa* and *Teucrium divaricatum*) to evaluate their potential responses to climate change and a consequent effect on their pollinators. Both species would handle moderate warming relatively well but would be negatively affected by strong warming predicted for the end of this century.

## Introduction

Global warming can have strong effects on plant species, their interactions and whole ecosystems (Tylianakis *et al*. 2008; Hegland *et al*. 2009; Traill *et al*. 2010). Elevated temperatures can, for instance, induce shifts in plant phenology across communities and change the interaction networks between plants and pollinators (Memmott *et al*. 2007; Hegland *et al*. 2009; Schweiger *et al*. 2010; Petanidou *et al*. 2014). One aspect of plant–pollinator interactions, which is particularly sensitive to climate change, is nectar production by plants (Scaven and Rafferty 2013). Plants have an optimum range of temperatures for nectar production, determined by their habitat and species characteristics (Jakobsen and Kristjánsson 1994). Often a moderate increase in average temperature can have a positive effect on plant nectar production (Pacini and Nepi 2007; Nocentini *et al*. 2013) but beyond that plants experience temperature stress, which can induce changes in nectar production (Petanidou and Smets 1996; Pacini *et al*. 2003; Scaven and Rafferty 2013). A number of studies from different systems have found decreased nectar volumes and nectar production rates at higher temperatures under both experimental and natural conditions (Jakobsen and Kristjánsson 1994; Petanidou and Smets 1996; Keasar *et al*. 2008). At the same time, nectar sugar concentration is usually less dependent on external factors and more constant throughout the day and the flowering season (Southwick 1983; Villarreal and Freeman 1990; Nocentini *et al*. 2013). The amount of sugar produced per flower has nevertheless been shown to depend on nectar volume rather than concentration (Torres and Galetto 1998; Hoover *et al*. 2012) and thus can be strongly affected by changes in ambient temperatures.

A decrease in nectar quantity and sugar content in response to global warming reduces the amount of resources available for pollinators and thus can have negative effects on plant–pollinator interactions (Hegland *et al*. 2009; Hoover *et al*. 2012; Scaven and Rafferty 2013). Under temperature stress plants can even begin to produce flowers without any nectar (Petanidou and Smets 1996), which further reduces the amount of resources. A decrease in floral rewards, but also in the variation of nectar production patterns within a plant, can change pollinator behaviour patterns (Real and Rathcke 1988; Zimmerman 1988). Most often, pollinators are found to be risk-sensitive to greater variability of nectar volume (Shafir 2000; Keasar *et al*. 2008) and high variation can therefore decrease plant attractiveness to pollinators (Zimmerman 1988). Consequently, climate change can reduce plant reproductive success due to changed interaction patterns with pollinators and possibly threaten the persistence of both plant and pollinator populations (Scaven and Rafferty 2013).

The Mediterranean Basin is considered to be one of Europe's regions most threatened by climate change, due to the elevated drought risk during summer (Giorgi 2006; Hickler *et al*. 2012), caused by elevated temperatures in combination with decreased precipitation during summer (Giorgi and Lionello 2008; IPCC 2013). Different climate scenarios predict 4–5 °C warming for summer months by the end of the century, relative to the end of the 20th century (Giorgi and Lionello 2008; IPCC 2013). These changes in temperature and precipitation regime can generate increasingly severe conditions for plant communities and consequently increase the vulnerability of mutualistic interaction networks in the Mediterranean region. In the water-limited phryganic communities (East-Mediterranean low scrub), the nectar production is generally low, with only a few species producing large quantities (i.e. >0.5 µL) of nectar per flower (Petanidou and Smets 1995). In these systems, Lamiaceae are a dominant plant group constituting the main source of water and nutrients for pollinators (Herrera 1985; Petanidou and Vokou 1993; Petanidou and Smets 1995; Petanidou 2007). Their role is particularly vital during summer, when nectar production in this system is challenged by high ambient temperatures, whereas nectar-feeding insect diversity is high (Petanidou and Vokou 1993; Petanidou *et al*. 1995).

In this study, we explore the effect of temperature on the nectar production of two common phryganic species of Lamiaceae visited predominantly by bees, both honeybees and wild bees. In both a climate-controlled and natural setting, we study the patterns of flower and nectar production and their variation in response to a wide range of temperatures, including temperatures higher than current climatic means, thus allowing us to investigate the potential effects of climate change on nectar rewards available to pollinators in the future. Our hypothesis is that strongly elevated temperatures reduce flower and nectar production and affect the variation in nectar production patterns. Such results could indicate a possible threat to pollinators and to the persistence of both plant and pollinator populations in the Mediterranean systems. It may also affect negatively bee-keeping and honey production, which play an important role in the economy of this area.

## Methods

### Study species

We tested the effect of temperature on the nectar production of two perennial Mediterranean species of the Lamiaceae family, *Ballota acetabulosa* (L.) Benth. and *Teucrium divaricatum* Sieber ex Heldr. Both species inhabit the same phryganic communities, but prefer to grow in different microhabitats. While *B. acetabulosa* often grows near stony structures or walls (e.g. abandoned agricultural terraces) or in places partially shaded by taller vegetation (e.g. in dehesa type scrub) (Petanidou *et al*. 2000; T. Petanidou, pers. obs.), *T. divaricatum* clearly prefers well-sunlit open habitats. Both species flower at the same time of the year, from May to July (Davis 1982; Petanidou 1991). They are visited by a wide range of medium- to large-sized wild bees of the families Megachilidae and Apidae and are also important food plants for bee-keeping (Petanidou 1991; Dauber *et al*. 2010; T. Petanidou *et al.* unpubl. data).

### Experiment design

Plants of *B. acetabulosa* used in the experiment were grown from seeds collected from the I. & A. Diomedes Botanical Garden of Athens University in 2005. Seeds were sown in October 2013, potted as seedlings, and grown outdoors until flowering. As for *T. divaricatum*, entire plants were collected from a natural population on Lesvos Island in October 2013, potted and grown outdoors until they were in bloom, ready to be used in the experiment.

The experiment was conducted at the University of the Aegean in Mytilene on Lesvos Island. Both study species were subjected to the same treatments simultaneously. For each species, two groups of plants were considered. One treatment group (15 plants per species) was tested under different temperature regimes in a climate chamber (Walk-in GRW-20 CMP 3/TBLIN, CDR Chryssagis™) and the second treatment group (six plants per species) was grown outdoors under naturally varying temperature and air humidity conditions. Comparing the two treatments allows us to estimate the effect of temperature separately from the effect of time and thus see, whether the trends are driven solely by temperature or whether they may be partially caused by the plants’ intrinsic limitations (e.g. exhausted nutrient reserves), which plants naturally incur during their flowering period. Some of the initial plants (one for *B. acetabulosa* in the climate chamber, and 16 for *T. divaricatum*, of which 11 in the climate chamber and 5 outdoors) were replaced with new ones during the experiment when they got close to the end of their flowering period, in order to have the same number of flowering plants at all tested temperatures. Altogether, including the replacement plants, 22 *B. acetabulosa* and 38 *T. divaricatum* plants were employed. Each plant was labelled with a unique number and considered as a separate entity in the subsequent analyses. The experiment for both species lasted from 24 May to 17 June 2014 in the climate chamber and in the outdoors from 24 May to 8 July 2014, following the plants through the flowering period both in the climate chamber and outdoors.

In the climate chamber, a 14-h photoperiod was used for both species, corresponding to the natural day length at the time of the year. A mixture of plant growth fluorescent lamps (Gro-lux) and low-pressure sodium lamps were used, with a total light intensity of ∼800 μmol m^−2^ s^−1^ (∼43 000 lx) over the waveband 400–700 nm in the chamber. Light intensity in the chamber was somewhat lower than what the plants would experience in nature; however, the intensity was constant throughout the days, compensating for the lower intensity.

The climate chamber experiment for both species was started at the lowest temperatures, and the temperature was increased every 3 days by 3 °C increments. Tested 24 h average temperatures ranged from 17.5 to 38.5 °C (day temperatures 20–41 °C, night temperatures always 6 °C lower, corresponding to the approximate natural difference between day and night temperatures at the time of the year). Tested temperatures were chosen to cover a range around the long-term (1958–2001) June average temperature of ∼26 °C according to Elefsis weather station near Athens, Greece, but including also elevated temperatures at least up to the temperatures predicted by climate change scenarios (IPCC 2013) to test for the effect of future climate warming. Relative air humidity was kept constant at 60 ± 5% during the day and 80 ± 5% during the night, corresponding approximately to current natural conditions. Plants were watered on the first day of every temperature step to avoid water stress.

Plants in the outdoor group were placed in an open area in full sunlight and were covered with large and airy tulle cages to prevent pollinator visits. The light intensity inside the covered cages was higher than in the climate chamber (∼1400 μmol m^−2^ s^−1^ or ∼75 000 lx). Data on ambient temperatures outdoors were obtained from the nearby (<300 m) climate station at the University of the Aegean in Mytilene (http://catastrophes.geo.aegean.gr/). The average temperature of the 3 days corresponding to each temperature step in the climate chamber was used in the analyses. The mean outdoor temperature during the test period ranged from 16.7 to 26.1 °C.

Nectar measurements both in the climate chamber and in the outdoor group were taken between 1230 and 1700 h on Day 3 of each temperature step, to give the plants in the climate chamber time to adjust to the changed conditions before sampling. Flowers were sampled on the first day of anthesis following Petanidou and Smets (1996). To ensure that only first day flowers were sampled, all open flowers were removed on Day 2, 24 h before nectar collection. Nectar was extracted from flowers with Drummond microcaps^®^ (2 and 1 μL for *B. acetabulosa*; 0.5 μL for *T. divaricatum*), using a single flower per sample. Three flowers per plant, selected randomly from among the flowers directly exposed to light, were sampled for nectar volume and sugar concentration per flower. Nectar sugar concentration of each of the sampled flowers was measured by using special hand refractometers for small nectar volumes (Bellingham & Stanley LTD, Tunbridge Wells). After sampling, the same three flowers per plant that were sampled for nectar were dried at 50 °C for 12 h and their biomass per flower was weighed (calyx and corolla together). All flowers, which had opened during the previous 24 h were counted and removed after nectar sampling on Day 3 before proceeding to the next temperature set.

### Data analysis

The sugar content per flower (micrograms) was calculated (volume per flower × concentration × density) with the density values taken from available sugar solution density tables (Dafni *et al*. 2005). In the very few cases (18/840 flowers of *B. acetabulosa* and 7/845 of *T. divaricatum*) when the collected nectar volume was too small for the refractometer to measure sugar concentration, the average value of sugar concentration of the other two flowers from the same plant was used, in order to be able to calculate sugar content per flower. For data analysis, we used the average values of the three sampled flowers per plant. Sugar content per plant was calculated by multiplying the average sugar content per flower with the number of flowers per plant produced on Day 3. The proportion of empty flowers per plant (i.e. producing no nectar) was also calculated based on the three flowers sampled for nectar. Proportion of empty flowers per plant was used in models only in the case of *B. acetabulosa*, because *T. divaricatum* produced only few nectarless flowers. We calculated the coefficient of variation (CV) for each of the measured flower traits (nectar volume, concentration, sugar content and biomass per flower) to characterize trait variation within a plant. In the case of sugar concentration per flower, the CV was determined based on the original data, prior to calculating the sugar concentration for the measurements with too small nectar volumes for direct sugar concentration detection. If the mean trait value of a plant was zero, the CV of the corresponding trait was also defined to be zero for that plant. Nectar volume per flower, sugar content per flower, sugar content per plant, number of flowers per plant and biomass per flower data were log-transformed and nectar sugar concentration per flower was logit-transformed to deal with the constraints of percentage data.

We tested for the possible differences between the original and replacement plants of *T. divaricatum* in their response to time, using linear mixed models (LMM) with interaction terms (‘time × replacement group’). There were no significant differences in the studied traits, except in sugar concentration per flower in the outdoor group (time^2^× replacement group interaction, *t* = −2.013, *P* = 0.049). Similarity of response indicates that the replacement plants had similar response patterns to the original plants and that the two plant groups can be analysed together in order to identify the general response patterns.

Data analysis was carried out in three consecutive steps. First, we used LMM models to identify the effect of temperature on plant traits in the climate chamber, testing both linear and quadratic effect of temperature on the traits and using plant number (plant ID) as a random factor. Analysis of variance (ANOVA) was used to compare the fit of the linear and quadratic models, based on the AIC values. However, it is essential to note that in the climate chamber we could not truly separate the effect of temperature and time on plants, since the temperature was increased linearly with time.

Secondly, we used the outdoor data to discern the separate effect of temperature on plant traits independent of time, fitting LMM models with two crossed random factors (plant ID and time from the beginning of the experiment) on the outdoor data. Naturally, temperature outdoors increased with time throughout the study period from May to July (*r* = 0.65, *P* = 0.009). However, this modelling approach enables us to separate the real effect of temperature on plant traits by taking into account the effect of time as a random factor.

As a third step, we identified the possible differences in response to time between the plants manipulated in the climate chamber and the plants grown outdoors. In these models we defined treatment group (climate chamber or outdoor) as an explanatory variable and used ‘trait × group’ interaction for identifying differences between the groups. No difference in trait response between the groups would therefore indicate that the trends in the climate chamber could be explained by the effect of time and that the effect of temperature was negligible or at least not significant. If the interaction terms in the models are significant, then the difference between the climate chamber and outdoor group can be attributed to the effect of elevated temperatures in the climate chamber. We did not perform the third set of models for the trait variation (CV) data because the effect of temperature on these traits was negligible in the first two sets of models.

In order to compare the response to time between the climate chamber and outdoor treatment groups, which had a different experiment duration, we used standardized time (mean = 0, SD = 1) in the models. Although having a different duration, the time span corresponds to the same ecological period for plants in both test groups, since the plants were examined from the beginning of full bloom until the end of the flowering period both in the climate chamber and outdoors.

All studied traits except the proportion of empty flowers per plant were modelled by following the description above. For modelling the proportion of empty flowers of *B. acetabulosa*, we followed a similar protocol, but used generalized linear mixed models (GLMM) with a negative binomial error distribution to account for the different distribution in the data. We also tested for the need to use zero-inflated models, but models with no zero-inflation parameter had a better fit (lower AIC values).

The analyses were conducted in R 3.1.1 (R Core Team 2014). Linear mixed models were fitted using the function *lmer* in the *lme4* package (Bates *et al*. 2014), the GLMM models were built using the function *glmmadmb* in the package *glmmADMB* (Fournier *et al*. 2012; Skaug *et al*. 2015). Additional *P*-values for the *t*-values in LMM models were calculated using the package *lmerTest* (Kuznetsova *et al*. 2014) and the conditional and marginal coefficients of determination (*R*^2^c and *R*^2^m) for the LMM models were calculated with the function *r.squaredGLMM* in the package *MuMIn* (Barton 2015). *R*^2^c shows the model variance explained by both fixed and random factors, while *R*^2^m represents the variance explained by fixed factors alone. Graphs were compiled using the function *ggplot* in the *ggplot2* package (Wickham 2009) using a smoothing function to plot the relationships.

## Results

### Ballota acetabulosa

**Figure 1.**
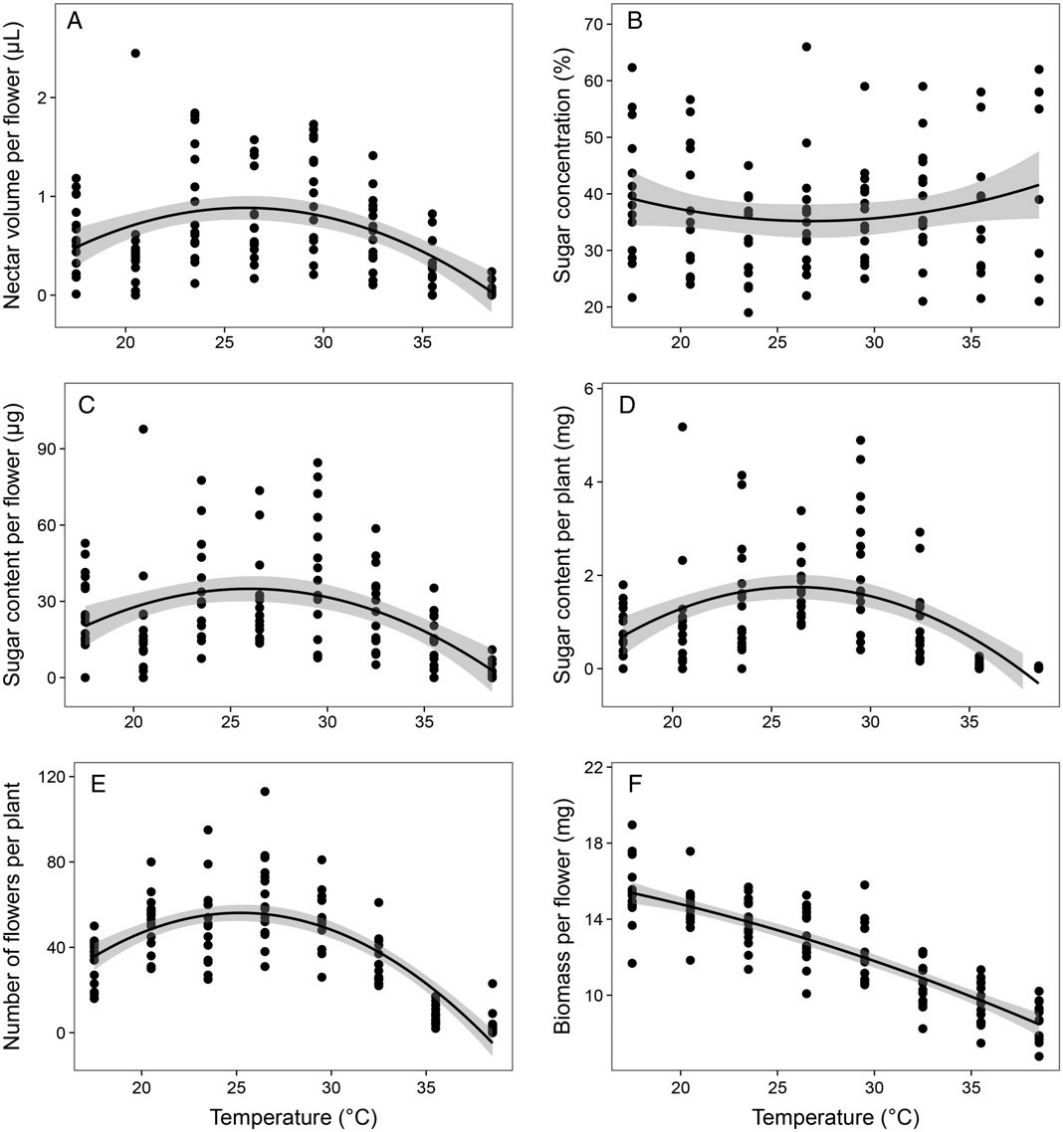
*Ballota acetabulosa* trait response to temperature in the climate chamber. Grey areas represent 95% confidence intervals.

In the climate chamber, the traits of *B. acetabulosa* showed a significant dependence on temperature through time (henceforth simply ‘temperature’; Table 1 and **[Supporting Information—Table S1]**). All tested traits had a positive unimodal relationship to temperature, except sugar concentration per flower, which showed a negative unimodal response (Table 1, Fig. 1). Optimum temperature for nectar volume per flower and sugar content per flower and per plant was at ∼25–26 °C (Fig. 1A, C and D), even though nectar sugar concentration per flower was the lowest at these temperatures (Fig. 1B). Number of flowers per plant per day was also highest at the same favourable temperatures (Fig. 1E), but biomass per flower declined unimodally through the test period (Fig. 1F). Contrarily, trait variation (CV) within a plant did not show significant changes in response to elevated temperatures in most of the tested traits, except CV of sugar concentration **[see Supporting Information—Table S2]**. Elevated temperatures in the climate chamber also accelerated flower production and thus resulted in shortening the flowering period nearly 2-fold compared with the outdoor group (duration of the experiment was 24 days in the climate chamber vs 45 days outdoors).

**Table 1.** Effect of temperature (simple and quadratic effect of temperature, ‘*T*’ and ‘*T*^2^’, respectively) on nectar and flower traits in the climate chamber. ‘*I*’ represents model intercept, ‘*R*^2^m’ and ‘*R*^2^c’ denote marginal and conditional coefficients of determination, indicating the variation explained by fixed factors (*R*^2^m) and the whole model (*R*^2^c; Barton 2015). Statistically significant (*P* < 0.05) results are presented in bold.

Species	Modelled trait		Estimate	SE	*t*	*P*	*R*^2^m	*R*^2^c
*B. acetabulosa*	Nectar volume per flower	*I*	−0.748	0.162	−4.614	**<0.001**	0.31	0.47
		*T*	0.079	0.012	6.441	**<0.001**		
		*T*^2^	−0.002	0.0002	−6.942	**<0.001**		
	Sugar concentration per flower	*I*	0.945	0.608	1.554	0.124	0.03	0.53
		*T*	−0.118	0.046	−2.586	**0.011**		
		*T*^2^	0.002	0.001	2.656	**0.009**		
	Sugar content per flower	*I*	−2.578	0.624	−4.134	**<0.001**	0.37	0.50
		*T*	0.320	0.047	6.863	**<0.001**		
		*T*^2^	−0.006	0.001	−7.477	**<0.001**		
	Sugar content per plant	*I*	−6.153	1.181	−5.208	**<0.001**	0.51	0.58
		*T*	0.740	0.088	8.389	**<0.001**		
		*T*^2^	−0.015	0.002	−9.239	**<0.001**		
	Number of flowers per plant	*I*	−1.436	0.321	−7.598	**<0.001**	0.82	0.84
		*T*	0.346	0.024	14.500	**<0.001**		
		*T*^2^	−0.007	0.0004	−16.561	**<0.001**		
	Biomass per flower	*I*	−1.815	0.046	−39.637	**<0.001**	0.75	0.92
		*T*	0.005	0.003	1.622	0.108		
		*T*^2^	−0.0003	0.0001	−5.194	**<0.001**		
*T. divaricatum*	Nectar volume per flower	*I*	−0.074	0.053	−1.383	0.170	0.08	0.41
		*T*	0.011	0.004	2.748	**0.007**		
		*T*^2^	−0.0002	0.0001	−2.428	**0.017**		
	Sugar concentration per flower	*I*	0.676	0.104	6.510	**<0.001**	0.02	0.53
		*T*	−0.005	0.004	−1.392	0.167		
	Sugar content per flower	*I*	0.088	0.257	0.341	0.734	0.08	0.60
		*T*	0.085	0.020	4.313	**<0.001**		
		*T*^2^	−0.002	0.0004	−4.055	**<0.001**		
	Sugar content per plant	*I*	−0.217	0.678	−0.320	0.749	0.42	0.82
		*T*	0.327	0.052	6.317	**<0.001**		
		*T*^2^	−0.007	0.001	−7.524	**<0.001**		
	Number of flowers per plant	*I*	−0.202	0.611	−0.330	0.742	0.54	0.87
		*T*	0.254	0.045	5.680	**<0.001**		
		*T*^2^	−0.006	0.001	−7.851	**<0.001**		
	Biomass per flower	*I*	−1.965	0.060	−32.871	**<0.001**	0.45	0.93
		*T*	0.004	0.005	0.934	0.353		
		*T*^2^	−0.0003	0.0001	−3.447	**<0.001**

Under this experimental design we were, however, unable to distinguish between the effect of temperature and time in the climate chamber as the relationships in this group could be due to both temperature and time. Under naturally varying conditions in the outdoor group we were able to model the effect of temperature separately, although the observed temperature range was narrower than the one tested in the climate chamber. For the outdoor group of *B. acetabulosa*, the linear models were favoured over the quadratic ones in all traits. Nevertheless, only sugar content per plant and biomass per flower had a significant relationship with temperature, showing a negative response to higher average temperatures (Table 2). Trait variation (CV) did not show a response to temperature, similarly to the climate chamber data, except in the case of CV of sugar concentration per flower, which was positively unimodally related to temperature (*R*^2^m = 0.15, *R*^2^c = 0.29) **[see Supporting Information—Table S3]**.

**Table 2.** Effect of temperature (simple and quadratic effect of temperature, ‘*T*’ and ‘*T*^2^’, respectively) on nectar and flower traits in the outdoor group. ‘*I*’ represents model intercept, ‘*R*^2^m’ and ‘*R*^2^c’ denote marginal and conditional coefficients of determination, indicating the variation explained by fixed factors (*R*^2^m) and the whole model (*R*^2^c; Barton 2015). Statistically significant (*P* < 0.05) results are presented in bold.

Species	Modelled trait		Estimate	SE	*t*	*P*	*R*^2^m	*R*^2^c
*B. acetabulosa*	Nectar volume per flower	*I*	0.819	0.317	2.583	**0.023**	0.14	0.63
		*T*	−0.027	0.014	−2.025	0.064		
	Sugar concentration per flower	*I*	−1.568	0.960	−1.634	0.126	0.03	0.27
		*T*	0.042	0.041	1.025	0.324		
	Sugar content per flower	*I*	3.416	1.076	3.175	**0.007**	0.13	0.53
		*T*	−0.093	0.046	−2.022	0.065		
	Sugar content per plant	*I*	8.532	2.690	3.172	**0.008**	0.23	0.75
		*T*	−0.262	0.115	−2.289	**0.041**		
	Number of flowers per plant	*I*	3.113	1.222	2.547	**0.022**	0.11	0.85
		*T*	−0.085	0.054	−1.587	0.134		
	Biomass per flower	*I*	−1.411	0.180	−7.854	**<0.001**	0.35	0.95
		*T*	−0.024	0.008	−3.058	**0.008**		
*T. divaricatum*	Nectar volume per flower	*I*	−0.073	0.197	−0.369	0.718	0.03	0.51
		*T*	0.009	0.008	1.006	0.333		
	Sugar concentration per flower	*I*	−0.231	0.788	−0.293	0.774	0.01	0.55
		*T*	0.015	0.034	0.450	0.660		
	Sugar content per flower	*I*	0.025	0.717	0.035	0.973	0.07	0.50
		*T*	0.053	0.031	1.743	0.113		
	Sugar content per plant	*I*	5.690	2.296	2.478	**0.028**	0.06	0.41
		*T*	−0.129	0.098	−1.313	0.213		
	Number of flowers per plant	*I*	3.803	1.124	3.383	**0.004**	0.15	0.57
		*T*	−0.107	0.049	−2.162	**0.048**		
	Biomass per flower	*I*	−3.375	0.441	−7.648	**<0.001**	0.09	0.75
		*T*	0.119	0.042	2.825	**0.021**		
		*T*^2^	−0.003	0.001	−2.727	**0.025**		

Comparison between the climate chamber and the outdoor group revealed a difference in the response to time in all *B. acetabulosa* traits, except sugar concentration per flower (Table 3 and **[Supporting Information—Fig. S1]**). The difference between the responses indicates an additional effect of temperature on plant traits in the climate chamber. Since the outdoor models indicated that temperature had no separate significant effect on nectar volume per flower, sugar content per flower and number of flowers per plant (Table 2), we can conclude that the difference observed between these trait responses is caused by the effect of elevated temperatures **[see Supporting Information—Fig. S1A, C and E]**.

**Table 3.** Comparison models testing the difference of the effect of time (simple and quadratic effect, ‘Time’ and ‘Time^2^’, respectively) on nectar and flower traits between the climate chamber and outdoor treatment (‘group’). Only interaction terms are presented here from the model full results. Statistically significant (*P* < 0.05) results are presented in bold.

Species	Modelled trait		*t*	*P*
*B. acetabulosa*	Nectar volume per flower	Time × group	−0.757	0.450
		Time^2^ × group	3.543	**<0.001**
	Sugar concentration per flower	Time × group	0.310	0.757
		Time^2^ × group	−1.841	0.069
	Sugar content per flower	Time × group	0.554	0.581
		Time^2^ × group	3.880	**0.002**
	Sugar content per plant	Time × group	−1.209	0.228
		Time^2^ × group	3.007	**0.003**
	Number of flowers per plant	Time × group	−3.824	**<0.001**
		Time^2^ × group	1.027	0.306
	Biomass per flower	Time × group	−4.379	**<0.001**
		Time^2^ × group	0.415	0.679
*T. divaricatum*	Nectar volume per flower	Time × group	0.481	0.632
		Time^2^ × group	−2.540	**0.012**
	Sugar concentration per flower	Time × group	−0.606	0.546
		Time^2^ × group	1.868	0.064
	Sugar content per flower	Time × group	1.058	0.292
		Time^2^ × group	−0.997	0.320
	Sugar content per plant	Time × group	−1.041	0.300
		Time^2^ × group	−0.066	0.948
	Number of flowers per plant	Time × group	−0.418	0.676
		Time^2^ × group	2.679	**0.008**
	Biomass per flower	Time × group	4.510	**<0.001**
		Time^2^ × group	3.622	**<0.001**

At the moderately warm temperatures in the climate chamber (∼26.5–29.5 °C) the plants had no empty flowers (producing no nectar) per plant, while at lower and higher temperatures the proportion of empty flowers increased. Nevertheless, modelling showed that the proportion of empty flowers per plant was not significantly related to temperature and the comparison between climate chamber and outdoor data was also not significant **[see Supporting Information—Table S4]**.

### Teucrium divaricatum

**Figure 2.**
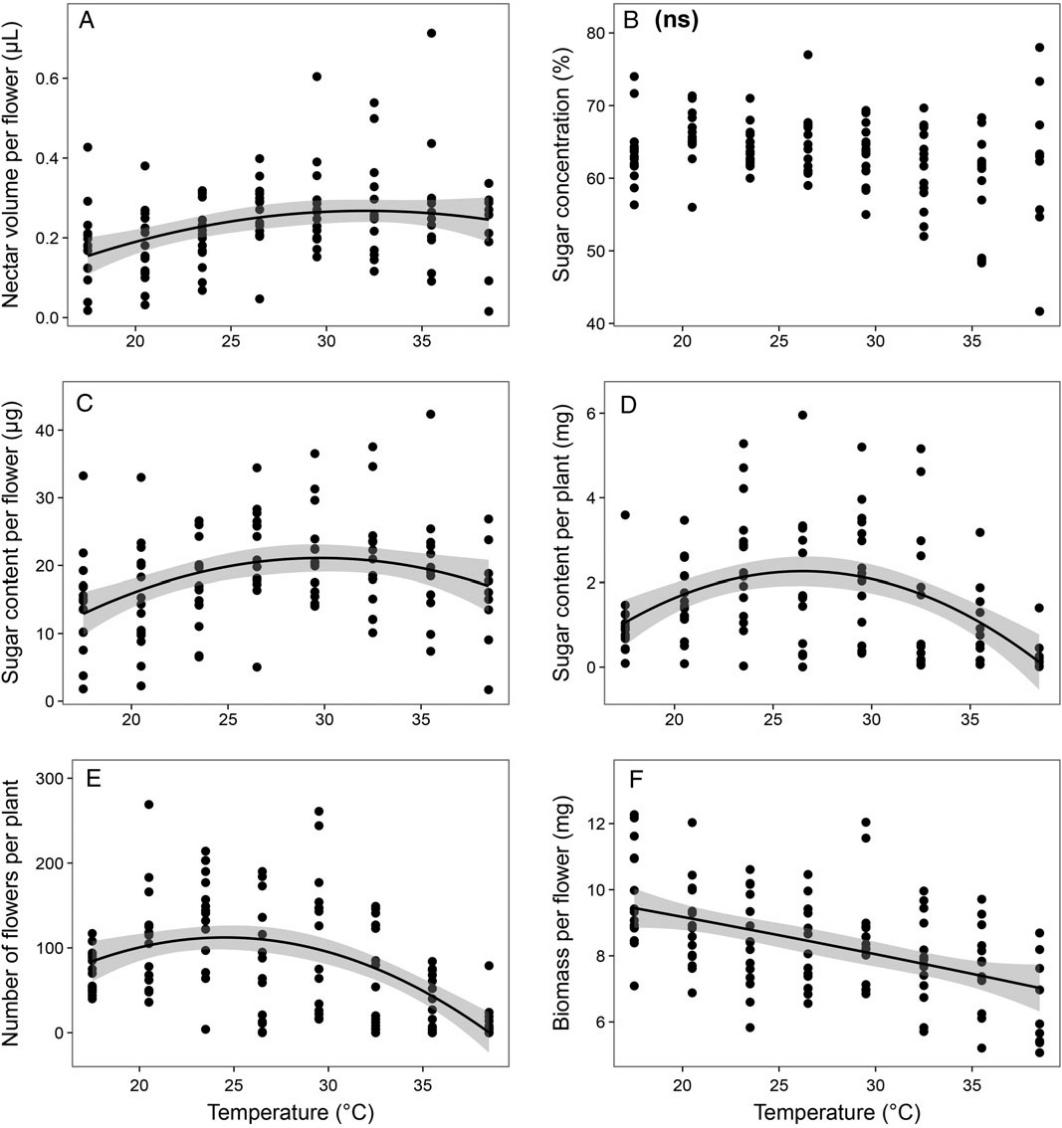
*Teucrium divaricatum* trait response to temperature in the climate chamber. Grey areas represent 95% confidence intervals. Non-significant (*P* > 0.05) relationships are marked with ‘ns’.

All tested traits of *T. divaricatum*, except sugar concentration per flower, were significantly positively unimodally related to temperature in combination with time in the climate chamber (Table 1, Fig. 2). Nectar volume and flower sugar content per flower of *T. divaricatum* peaked at higher average temperatures (∼29–33 °C; Fig. 2A and C) than the number of flowers per plant produced per day (∼24–25 °C; Fig. 2E). Sugar content per plant was therefore greatest at ∼26–27 °C (Fig. 2D). Trait variation (CV) of flower traits was not related to elevated temperatures in most of the studied traits, except CV of sugar concentration [**see Supporting Information—Table S2**]. In the outdoor group, only two traits showed a significant relationship to average temperature; the number of flowers per plant decreased with increasing temperatures and biomass per flower was positively unimodally related to temperature (Table 2). Most of the outdoor models also had very low marginal coefficients of determination (*R*^2^m), showing that much of the variation was explained by differences between individual plants and not by temperature (Table 2).

Comparing the response between the climate chamber and the outdoor group in response to time showed a difference in nectar volume per flower, number of flowers per plant and biomass per flower (Table 3 and **[Supporting Information—Fig. S2]**). Number of flowers per plant and biomass per flower also showed a separate response to temperature in the outdoor models (Table 2), thus it is more difficult to say how much of the difference could be caused by the elevated temperatures in the climate chamber **[see Supporting Information—Fig. S2E and F]**. However, the difference in the case of nectar volume per flower is likely caused by the effect of elevated temperatures in the climate chamber **[see Supporting Information—Fig. S2A]**.

## Discussion

### Nectar secretion in the Mediterranean under global warming

We studied the effect of a range of temperatures on two phryganic Lamiaceae species in Greece to evaluate the potential threat of climate change to floral nectar production and plant–pollinator interactions in the Mediterranean. The Mediterranean region is considered to be particularly threatened by global warming in the future due to strongly elevated temperatures and a heightened probability of drought during the summer (Giorgi 2006; Hickler *et al*. 2012). The average summer temperatures across the Mediterranean between 1961 and 1990 were between ∼22.5 and 30 °C (New *et al*. 1999) but the climate warming scenarios now predict a 0.9–1.2 °C of warming for the summer months over the next two decades and up to 4–5 °C temperature increase before the end of the century (Giorgi and Lionello 2008; Giannakopoulos *et al*. 2009; IPCC 2013).

Strongly elevated temperatures, predicted to prevail in the Mediterranean region in the end of the century, would likely decrease nectar secretion and sugar production in both *B. acetabulosa* and *T. divaricatum*, which based on our results, currently grow in the wild at or close to their optimum temperature range for nectar secretion. This effect can be particularly strong in regions of the Mediterranean where the current average temperatures are higher. Strongly elevated temperatures in the climate chamber decreased sugar content per plant of both *B. acetabulosa* and *T. divaricatum* (Figs 1D and 2D) by decreasing the volume of nectar produced per flower (Figs 1C and 2C) and the number of flowers per plant (Figs 1E and 2E). High temperatures induce physiological stress in plants, which decreases nectar production per flower (Petanidou and Smets 1996; Pacini and Nepi 2007; Scaven and Rafferty 2013) and can cause plants to produce fewer flowers (Saavedra *et al*. 2003). Extreme climate warming could consequently have a negative effect on nectar production and hence on the phryganic communities, where Lamiaceae are a dominant plant group (Petanidou and Vokou 1993).

Moderate warming predicted for the next two decades, however, should have only a moderate effect on *B. acetabulosa* nectar secretion, remaining close to this species’ optimum range, and even promote nectar production of *T. divaricatum*, which demonstrated a relatively high optimal temperature for nectar volume per flower (30–35 °C; Fig. 2A) and sugar content per flower (∼29 °C; Fig. 2C). Similarly high temperature optimum (32.5 °C during the day, analogous to the optimal daily temperatures of *T. divaricatum*) has also been found for the sugar content per flower of *Thymus capitatus*, another phryganic species (Petanidou and Smets 1996). In species with similarly high temperature optima, climate change can potentially increase nectar production, at least in the case of moderate warming. However, sugar content per plant of *T. divaricatum* peaked at lower temperatures (optimum ∼26–27 °C; Fig. 2D) than sugar content per flower, indicating that the nectar production per plant could still be negatively affected by even moderate warming, even if sugar content per flower can still increase under higher temperatures. The effect of moderate warming on the sugar content per plant of *T. divaricatum*, as well as *B. acetabulosa* sugar content per flower (Fig. 1C) and sugar content per plant (optimum ∼25–27 °C; Fig. 1D), might in the near future be promoted in some regions of the Mediterranean with lower average temperatures, or be moderately decreased in regions with higher temperatures.

In contrast to nectar and sugar production, trait variation (CV) within a plant was not related to temperature in most of the tested traits. Only CV of sugar concentration was positively related to temperature indoors (in both species) and positively unimodally related to temperature outdoors (in *B. acetabulosa*). Changes in the variation of nectar production can decrease plant attractiveness to pollinators (Real and Rathcke 1988; Zimmerman 1988; Shafir 2000), thus having a negative impact on plant pollination and consequently on plant reproduction and population persistence (Scaven and Rafferty 2013). Nevertheless, the stability of trait variation patterns in our experiment signifies that in these two species this aspect of plant–pollinator interactions could be relatively unaffected by elevated temperatures and not cause additional alterations under climate warming.

Moderate warming can be beneficial for nectar production at least as long as the plants are not water stressed. The Mediterranean plants are indeed well adapted to cope with the hot and dry conditions during the flowering period (Petanidou 2007). However, different scenarios of climate change predict a substantial decrease in summer precipitation in the Mediterranean region in addition to increased temperatures (Giorgi and Lionello 2008; Giannakopoulos *et al*. 2009; IPCC 2013). Consequently, the actual effect of climate warming with additional water stress (Villarreal and Freeman 1990; Carroll *et al*. 2001) could further increase the negative effect of elevated temperatures on nectar production in the phryganic systems. The combined effect of temperature and drought on plants and their ability to adapt to future climate changes in this region still needs further study.

### Differences in species' response to global warming

Our two study species responded to the elevated temperatures somewhat differently, which suggests possible disparate responses to climate change in phryganic species. For one, *B. acetabulosa* optimal temperatures for nectar and sugar production were in general lower, close to the current average temperatures, and the species therefore more sensitive to climate warming than *T. divaricatum*, which had higher optimal temperatures for nectar secretion (Figs 1 and 2). Additionally, several traits of *B. acetabulosa* exhibited dependence on elevated temperatures (nectar volume per flower, sugar content per flower and number of flowers per plant) in addition to the effect of time through the flowering period. On the other hand, in *T. divaricatum* it was only nectar volume per flower that clearly depended on elevated temperatures. Strong dependence on temperature implies that *B. acetabulosa* could be rather sensitive to temperature changes, whereas *T. divaricatum* might be more moderately affected by climate warming.

The differences in species' nectar production patterns could be caused by the plants' adaptations to the different phryganic microhabitats, which the two species inhabit—*B. acetabulosa* prefers modified or somewhat protected microhabitats (understorey, partially shaded, neighbouring to different structures), while *T. divaricatum* grows under full sun (Petanidou and Smets 1996; Petanidou *et al*. 2000). As a plant of open natural areas, *T. divaricatum* is adapted to particularly high temperatures and does not experience physiological stress at moderately higher than average temperatures, akin to other open phryganic species, such as *Thymus capitatus*, which has a similarly high optimal temperature range for nectar sugar production (Petanidou and Smets 1996). At the same time, *B. acetabulosa* as a plant of less exposed habitats could be less adapted to the heat and considerably more sensitive to temperature rise. This difference suggests a stronger effect of climate change on understorey plants in the phryganic systems, which are less adapted to high temperatures and the effect of drought. At the same time, species that are adapted to the harsh conditions of the open phrygana might be able to cope better with the forthcoming changes.

### Effect of global warming on Mediterranean pollinators

Our results demonstrate that in case of moderate warming, as predicted in the Mediterranean region for the next two decades, nectar sugar production and consequently the amount of resources available for pollinators could be moderately adversely affected in the case of some Mediterranean species (e.g. *B. acetabulosa*) and might even be benefitted in others (e.g. *T. divaricatum*). This difference in species responses suggests that at the community level species nectar production might be able to balance out, at least for the more generalized pollinators (Scaven and Rafferty 2013). Nevertheless, in the case of more extreme warming, as predicted for the end of the century, nectar production of both species is expected to decrease.

Both our study species are among the highest nectar producers in phrygana (Petanidou and Smets 1995) and are therefore an essential resource for a number of pollinator species (Petanidou and Vokou 1993; T. Petanidou *et al*., unpubl.). Due to the greater sensitivity of *B. acetabulosa* to elevated temperatures, the effect of climate warming could be more pronounced on the pollinators of this species already during the next few decades under the effect of moderate warming. Although plant–pollinator networks, especially generalist interactions, are expected to be rather robust to the effect of climate change (Devoto *et al*. 2007; Schweiger *et al*. 2010; DeLucia *et al*. 2012; Burkle *et al*. 2013), the loss of a generalist plant species can still be a considerable risk for the population persistence of pollinators (Memmott *et al*. 2004). Even if the plant and pollinator populations are able to persist for some time, the strength of interactions can be changed due to alterations in plant resources and pollinator behaviour and thus still significantly affect the mutualistic interaction networks due to climate warming (Memmott *et al*. 2007; Scaven and Rafferty 2013).

Moreover, experimental warming in the climate chamber accelerated plant flowering and thus reduced the length of flowering period under elevated temperatures. Under natural conditions, shorter flowering time in consequence of global warming could increase the probability of creating temporal mismatches with pollinators (Memmott *et al*. 2007; Hegland *et al*. 2009; DeLucia *et al*. 2012). Since Lamiaceae are the most essential group for the pollinators in the phryganic systems in summer (Herrera 1985; Petanidou and Smets 1995; Petanidou 2007), the reduced nectar secretion and temporal mismatches with these species at strongly elevated temperatures could have a considerable impact on the phryganic pollinator fauna. In addition, it could also negatively affect apiculture in the Mediterranean region, which in the phryganic systems is strongly dependent on the abundance and nectar production of different Lamiaceae species (Petanidou and Smets 1995).

## Conclusions

Mediterranean ecosystems may be able to endure moderate climate warming without major changes in plant–pollinator interactions, at least as long as the plant communities are not overly water stressed. Additional water stress due to decreased rainfall predicted by climate change scenarios could, however, induce stronger and more rapid changes in nectar production and plant–pollinator interactions. More extensive changes in Mediterranean communities can be expected towards the end of the century due to more extreme warming, when even species adapted to the severe conditions of open phrygana, such as *T. divaricatum*, might be excessively stressed by the elevated temperatures. Consequent changes in plant–pollinator interactions can include weakening or disruption of interaction networks and can eventually lead to a possible loss of pollinator species in the Mediterranean systems (Hegland *et al*. 2009; Scaven and Rafferty 2013; Petanidou *et al*. 2014).

## Sources of Funding

The research has been co-financed by the European Union (European Social Fund—ESF) and Greek national funds through the Operational Program ‘Education and Lifelong Learning’ of the National Strategic Reference Framework (NSRF)—Research Funding Program: THALES. Investing in knowledge society through the European Social Fund.

## Contributions by the Authors

T.P. conceived the idea, P.T. and K.T. conducted the experiment, T.P. and T.T. supervised and supported the experimental work. K.T. analysed the data and led the writing with the assistance of T.P. and T.T.

## Conflict of Interest Statement

None declared.

## Supporting Information

The following additional information is available in the online version of this article –

**Table S1.** Nectar secretion values of the study plants under different temperature regimes in the climate chamber.

**Table S2.** Effect of temperature on the CV of flower traits in the climate chamber.

**Table S3.** Effect of temperature on the CV of flower traits in the outdoor group.

**Table S4.** Proportion of empty flowers of *Ballota acetabulosa* in relation to temperature and comparison models testing the difference of the effect of time between the climate chamber and the outdoor treatment.

**Figure S1.** Comparison of *Ballota acetabulosa* trait response to time between the climate chamber and outdoor group.

**Figure S2.** Comparison of *Teucrium divaricatum* trait response to time between the climate chamber and outdoor group.

Additional Information
