# Supplementary material for: Climate change reduces nectar secretion in two common Mediterranean plants
Source: AoB Plants. 2015 Sep 15;7:plv111. doi: 10.1093/aobpla/plv111 (PMC4614813; doi:10.1093/aobpla/plv111)
Supplement: Additional Information [file supp_7_plv111_index.html]

Climate change reduces nectar secretion in two common Mediterranean plants — Climate change reduces nectar secretion in two common Mediterranean plants — Additional Information 

# Climate change reduces nectar secretion in two common Mediterranean plants

## Additional Information

Additional Information

- Additional Information - Doc file
