# Supplementary material for: Climate change reduces nectar secretion in two common Mediterranean plants
Source: AoB Plants. 2015 Sep 15;7:plv111. doi: 10.1093/aobpla/plv111 (PMC4614813; doi:10.1093/aobpla/plv111)
Supplement: Additional Information [file supp_plv111_plv111supp.doc]

**Supporting Information**

**Table S1.** Nectar secretion values of the study plants under different temperature regimes in the climate chamber. Presented values are means over all sampled plants (±SE, number of plants).

| Time (days since start of experiment) | Average temperature (°C) | Nectar volume per flower (μl) | | | Sugar concentration per flower (%) | | | Sugar content per flower (μg) | | | Sugar content per plant (mg) | | | Number of flowers per plant | | | Percentage of empty flowers per plant (%) | | | Biomass per flower (mg) | | |  | |
| --- | --- | --- | --- | --- | --- | --- | --- | --- | --- | --- | --- | --- | --- | --- | --- | --- | --- | --- | --- | --- | --- | --- | --- | --- |
| *Ballota acetabulosa* | | |  | | |  | | |  | | |  | | |  | | |  | | |  | | |  |
| 3 | 17.5 | 0.62±0.09, 15 | | | 40.1±3.12, 14 | | | 26.6±3.87, 15 | | | 0.85±0.14, 15 | | | 33.3±2.66, 15 | | | 4.5±4.45, 15 | | | 15.4±0.44, 15 | | |  | |
| 6 | 20.5 | 0.46±0.15, 15 | | | 37.6±3.20, 13 | | | 19.6±6.21, 15 | | | 1.04±0.33, 15 | | | 51.4±3.44, 15 | | | 26.7±9.87, 15 | | | 14.6±0.31, 15 | | |  | |
| 9 | 23.5 | 0.95±0.15, 15 | | | 31.6±2.02, 14 | | | 33.7±5.51, 14 | | | 1.53±0.32, 15 | | | 50.5±4.93, 15 | | | 2.2±2.22, 15 | | | 13.8±0.32, 15 | | |  | |
| 12 | 26.5 | 0.78±0.12, 15 | | | 36.0±2.76, 15 | | | 30.6±4.53, 15 | | | 1.73±0.18, 15 | | | 63.1±5.35, 15 | | | 0±0, 15 | | | 13.0±0.38, 15 | | |  | |
| 15 | 29.5 | 1.02±0.14, 15 | | | 36.8±2.22, 15 | | | 42.3±6.31, 15 | | | 2.27±0.36, 15 | | | 54.8±3.54, 15 | | | 0±0, 15 | | | 12.3±0.40, 15 | | |  | |
| 18 | 32.5 | 0.65±0.10, 15 | | | 38.6±2.54, 15 | | | 26.8±4.11, 15 | | | 0.97±0.22, 15 | | | 32.5±2.81, 15 | | | 11.1±4.20, 15 | | | 10.6±0.30, 15 | | |  | |
| 21 | 35.5 | 0.31±0.07, 15 | | | 36.9±3.28, 12 | | | 13.3±2.86, 15 | | | 0.10±0.03, 15 | | | 8.0±1.13, 15 | | | 28.9±9.12, 15 | | | 9.5±0.29, 15 | | |  | |
| 24 | 38.5 | 0.05±0.02, 12 | | | 41.4±6.40, 7 | | | 2.5±1.03, 12 | | | 0.01±0.007, 12 | | | 3.6±1.50, 12 | | | 52.8±13.30, 15 | | | 8.7±0.30, 12 | | |  | |
| *Teucrium divaricatum* | | | |  | | |  | | |  | | |  | | |  | | |  | | |  | | |
| 3 | 17.5 | 0.18±0.03, 15 | | | 63.6±1.15, 15 | | | 14.3±1.96, 15 | | | 1.03±0.21, 15 | | | 71.5±6.38, 15 | | | 4.5±4.45, 15 | | | 9.7±0.39, 15 | | |  | |
| 6 | 20.5 | 0.17±0.02, 15 | | | 66.0±0.94, 15 | | | 15.1±2.06, 15 | | | 1.59±0.23, 15 | | | 111.1±15.80, 15 | | | 2.2±2.22, 15 | | | 8.9±0.34, 15 | | |  | |
| 9 | 23.5 | 0.20±0.02, 15 | | | 64.3±0.72, 15 | | | 17.0±1.58, 15 | | | 2.48±0.38, 15 | | | 137.9±15.26, 15 | | | 4.4±3.03, 15 | | | 8.4±0.37, 15 | | |  | |
| 12 | 26.5 | 0.26±0.02, 14 | | | 64.8±1.22, 14 | | | 22.0±1.91, 14 | | | 2.08±0.44, 14 | | | 85.8±17.59, 15 | | | 0±0, 14 | | | 8.2±0.34, 14 | | |  | |
| 15 | 29.5 | 0.27±0.03, 15 | | | 63.2±1.05, 15 | | | 21.7±1.67, 15 | | | 2.21±0.39, 15 | | | 110.2±21.01, 15 | | | 4.4±3.03, 15 | | | 8.6±0.39, 15 | | |  | |
| 18 | 32.5 | 0.27±0.03, 14 | | | 61.3±1.45, 14 | | | 20.8±2.12, 14 | | | 1.62±0.46, 14 | | | 64.6±15.35, 15 | | | 4.8±3.23, 14 | | | 7.9±0.35, 14 | | |  | |
| 21 | 35.5 | 0.34±0.07, 13 | | | 57.6±2.49, 13 | | | 21.9±3.00, 13 | | | 0.85±0.26, 13 | | | 34.1±8.30, 15 | | | 2.6±2.56, 13 | | | 7.7±0.36, 13 | | |  | |
| 24 | 38.5 | 0.22±0.04, 9 | | | 62.2±3.58, 9 | | | 15.8±2.50, 9 | | | 0.30±0.14, 9 | | | 10.9±5.24, 15 | | | 3.7±3.70, 9 | | | 6.6±0.45, 9 | | |  | |

**Table S2.** Effect of temperature (‘T’) on the CV of flower traits in the climate chamber. Both simple and quadratic effect of temperature were tested but linear models were better in all cases according to model AIC values. ‘I’ represents model intercept, ‘*R^2^m*’ and ‘*R^2^c*’ denote marginal and conditional coefficients of determination, indicating the variation explained by fixed factors (*R^2^m*) and the whole model (*R^2^c*; Barton, 2015). Statistically significant (p<0.05) results are presented in bold.

| Species | Modelled trait |  | Estimate | *SE* | *t* | *P* | *R^2^m* | *R^2^c* |
| --- | --- | --- | --- | --- | --- | --- | --- | --- |
| *Ballota acetabulosa* | |  |  |  |  |  |  |  |
|  | CV of nectar volume per flower | I | 0.664 | 0.166 | 4.014 | **<0.001** | 0.001 | 0.03 |
|  |  | T | -0.002 | 0.006 | -0.284 | 0.777 |  |  |
|  | CV of sugar concentration per flower | I | 0.080 | 0.048 | 1.673 | 0.098 | 0.04 | 0.12 |
|  |  | T | 0.004 | 0.002 | 2.179 | **0.032** |  |  |
|  | CV of sugar content per flower | I | 0.666 | 0.155 | 4.287 | **<0.001** | 0.003 | 0.03 |
|  |  | T | -0.003 | 0.005 | -0.600 | 0.550 |  |  |
|  | CV of biomass per flower | I | 0.027 | 0.015 | 1.752 | 0.083 | 0.02 | 0.02 |
|  |  | T | 0.001 | 0.001 | 1.674 | 0.097 |  |  |
| *Teucrium divaricatum* | |  |  |  |  |  |  |  |
|  | CV of nectar volume per flower | I | 0.357 | 0.132 | 2.714 | **0.008** | 0.004 | 0.39 |
|  |  | T | 0.003 | 0.005 | 0.633 | 0.528 |  |  |
|  | CV of sugar concentration per flower | I | 0.009 | 0.019 | 0.472 | 0.638 | 0.07 | 0.07 |
|  |  | T | 0.002 | 0.001 | 2.745 | **0.007** |  |  |
|  | CV of sugar content per flower | I | 0.358 | 0.129 | 2.785 | **0.007** | 0.002 | 0.37 |
|  |  | T | 0.002 | 0.004 | 0.443 | 0.659 |  |  |
|  | CV of biomass per flower | I | 0.053 | 0.022 | 2.391 | **0.019** | 0.02 | 0.17 |
|  |  | T | 0.001 | 0.001 | 1.328 | 0.188 |  |  |

**Table S3.** Effect of temperature (simple and quadratic effect of temperature, ‘T’ and ‘T2’, respectively) on the CV of flower traits in the outdoor group. ‘I’ represents model intercept, ‘*R^2^m*’ and ‘*R^2^c*’ denote marginal and conditional coefficients of determination, indicating the variation explained by fixed factors (*R^2^m*) and the whole model (*R^2^c*; Barton, 2015). Statistically significant (p<0.05) results are presented in bold.

| Species | Modelled trait |  | Estimate | *SE* | *t* | *P* | *R^2^m* | *R^2^c* |
| --- | --- | --- | --- | --- | --- | --- | --- | --- |
| *Ballota acetabulosa* | |  |  |  |  |  |  |  |
|  | CV of nectar volume per flower | I | 0.178 | 0.988 | 0.180 | 0.861 | 0.04 | 0.55 |
|  |  | T | 0.024 | 0.042 | 0.561 | 0.587 |  |  |
|  | CV of sugar concentration per flower | I | -5.738 | 1.970 | -2.913 | **0.005** | 0.15 | 0.29 |
|  |  | T | 0.490 | 0.168 | 2.910 | **0.005** |  |  |
|  |  | T^2^ | -0.010 | 0.004 | -2.842 | **0.006** |  |  |
|  | CV of sugar content per flower | I | 0.484 | 0.552 | 0.877 | 0.396 | 0.06 | 0.33 |
|  |  | T | 0.012 | 0.024 | 0.502 | 0.624 |  |  |
|  | CV of biomass per flower | I | 0.009 | 0.032 | 0.282 | 0.779 | 0.02 | 0.03 |
|  |  | T | 0.002 | 0.001 | 1.348 | 0.182 |  |  |
| *Teucrium divaricatum* | |  |  |  |  |  |  |  |
|  | CV of nectar volume per flower | I | 1.348 | 0.869 | 1.551 | 0.146 | 0.02 | 0.11 |
|  |  | T | -0.035 | 0.037 | -0.948 | 0.361 |  |  |
|  | CV of sugar concentration per flower | I | -0.008 | 0.148 | -0.051 | 0.959 | 0.02 | 0.16 |
|  |  | T | 0.004 | 0.006 | 0.622 | 0.536 |  |  |
|  | CV of sugar content per flower | I | 0.472 | 0.364 | 1.299 | 0.224 | 0.01 | 0.03 |
|  |  | T | 0.0002 | 0.016 | 0.013 | 0.990 |  |  |
|  | CV of biomass per flower | I | -0.024 | 0.056 | -0.425 | 0.676 | 0.08 | 0.47 |
|  |  | T | 0.004 | 0.003 | 1.785 | 0.091 |  |  |

**Table S4.** Proportion of empty flowers of *Ballota acetabulosa* in relation to temperature (linear and quadratic effect tested) and comparison models testing the difference of the effect of time between the climate chamber and the outdoor treatment (‘group’). Model standard errors (‘*SE’*) and *z*-values are presented (‘*z*’).

| Model | Fixed factors | Estimate | *SE* | *z* | *P* |
| --- | --- | --- | --- | --- | --- |
| *Climate chamber (random factors: plant ID)* | | | | |  |
|  | Intercept | 16.181 | 9.855 | 1.64 | 0.10 |
|  | temperature | -1.296 | 0.748 | -1.73 | 0.08 |
|  | temperature^2^ | 0.025 | 0.013 | 1.91 | 0.06 |
| *Outdoors (random factors: plant ID, time)* | | | | |  |
|  | Intercept | 219.378 | 139.350 | 1.57 | 0.12 |
|  | temperature | -18.908 | 11.837 | -1.60 | 0.11 |
|  | temperature^2^ | 0.406 | 0.250 | 1.62 | 0.10 |
| *Climate chamber vs outdoors (random factor: plant ID)* | | | | | |
|  | Intercept | 0.738 | 0.853 | 0.86 | 0.39 |
|  | group | 0.352 | 0.395 | 0.89 | 0.37 |
|  | time | -0.203 | 1.758 | -0.12 | 0.91 |
|  | time^2^ | 1.547 | 0.790 | 1.96 | 0.05 |
|  | time×group | 1.274 | 1.545 | 0.82 | 0.41 |
|  | time^2^×group | -0.039 | 1.986 | -0.02 | 0.98 |

**Figure S1.** Comparison of *Ballota acetabulosa* trait response to time between the climate chamber and outdoor group. Filled circles and solid lines denote measurements and the fitted smoothing function in the climate chamber, empty circles and dotted lines indicate the measurements and the fitted smoothing function outdoors. Grey areas represent 95% confidence intervals. Non-significant (p>0.05) differences between the responses are marked with ‘ns’.

**
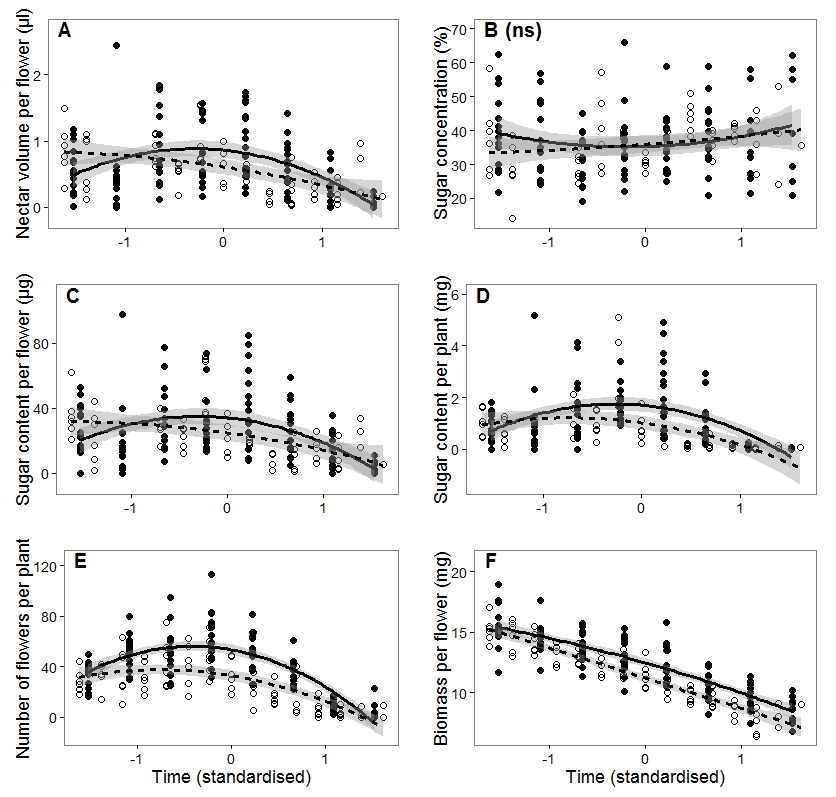
**

**Figure S2.** Comparison of *Teucrium divaricatum* trait response to time between the climate chamber and outdoor group. Filled circles and solid lines denote measurements and the fitted smoothing function in the climate chamber, empty circles and dotted lines indicate the measurements and the fitted smoothing function outdoors. Grey areas represent 95% confidence intervals. Non-significant (p>0.05) differences between the responses are marked with ‘ns’.

**
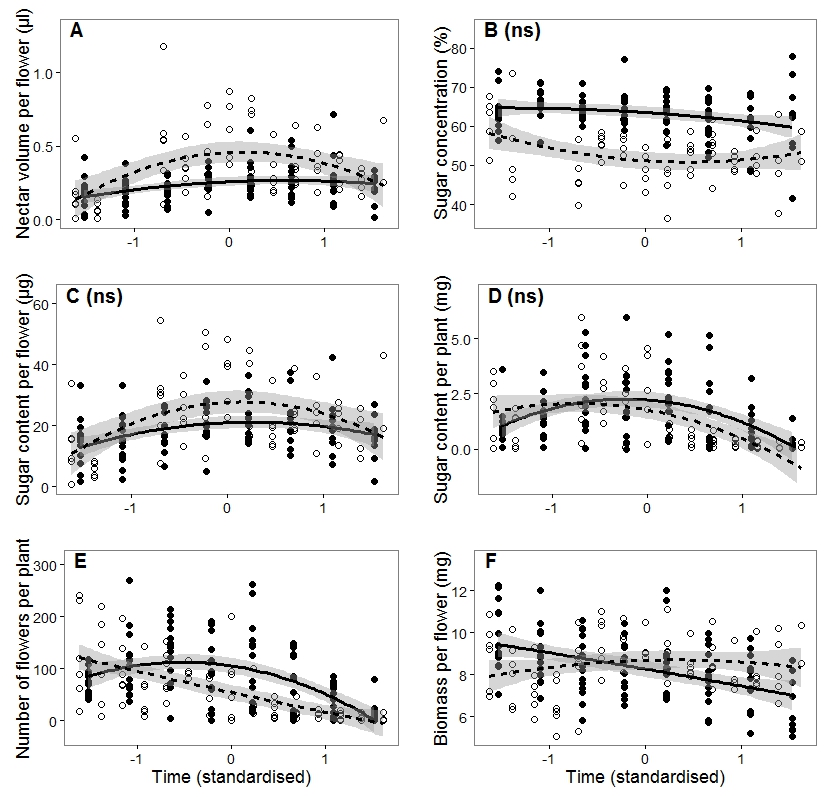
**
